# Supplementary material for: Origin and functional impact of early nonlinearities in primate retina
Source: bioRxiv. 2026 Mar 23:2026.03.19.713068. Preprint. [Version 1] doi: 10.64898/2026.03.19.713068 (PMC13041967; doi:10.64898/2026.03.19.713068)
Supplement: 1 [file NIHPP2026.03.19.713068v1-supplement-1.pdf]

bioRxiv preprint doi: https://doi.org/10.64898/2026.03.19.713068; this version posted March 23, 2026. The copyright holder for this preprint (which was not certified by peer review) is the author/funder, who has granted bioRxiv a license to display the preprint in perpetuity. It is made available under aCC-BY 4.0 International license.

Supplementary Figures

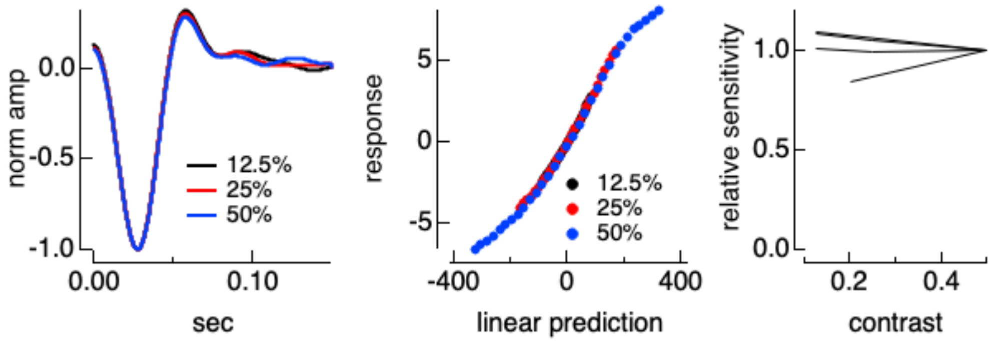

Figure S1: Little or no contrast adaptation. LN model fits to responses of a horizontal cell to Gaussian noise at the three contrasts indicated. Right panel shows the sensitivity, measured from the slope of the nonlinearity, for three such experiments.

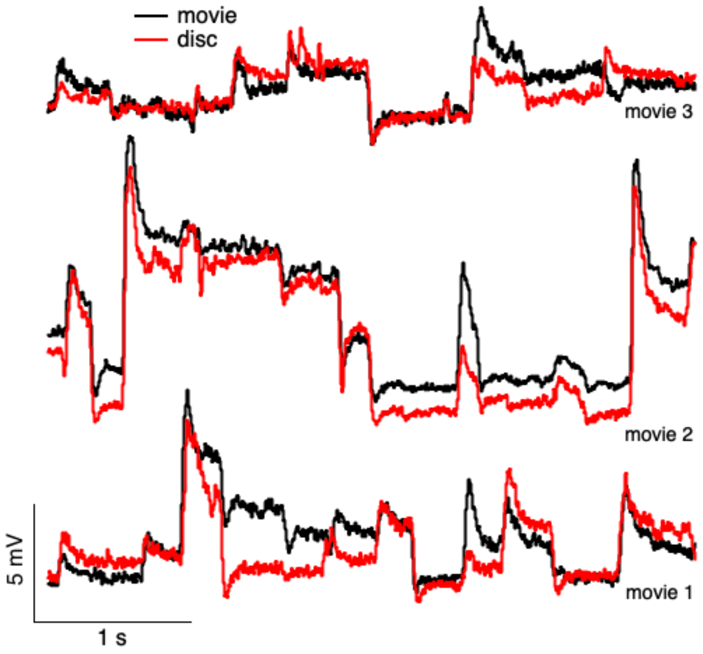

Figure S2: Horizontal cell responses to linear equivalent (red) and Doves (black) movies.
